# The seroconversion rate of QuantiFERON-TB Gold In-Tube test in psoriatic patients receiving secukinumab and ixekizumab, the anti-interleukin-17A monoclonal antibodies

**DOI:** 10.1371/journal.pone.0225112

**Published:** 2019-12-27

**Authors:** Chen-Yu Wu, Hsien-Yi Chiu, Tsen-Fang Tsai

**Affiliations:** 1 Department of Dermatology, Cathay General Hospital, Taipei, Taiwan; 2 Department of Dermatology, National Taiwan University Hospital and National Taiwan University College of Medicine, Taipei, Taiwan; 3 Department of Dermatology, National Taiwan University Hospital Hsin-Chu Branch, Hsinchu, Taiwan; University of Cape Town, SOUTH AFRICA

## Abstract

**Background:**

For psoriatic patients receiving biologics, the concern of tuberculosis (TB) infection exists. Although the TB risk of anti-interleukin (IL)-17A agents is generally considered very low, more real-world data are needed to support the safety.

**Objectives:**

This study aims to provide the real-world experience of using serial QuantiFERON-TB Gold In-Tube (QFT-GIT) test among patients treated with secukinumab or ixekizumab in Taiwan, an intermediate TB burden country, for the detection of latent TB infection (LTBI) reactivation or newly acquired TB infection.

**Methods:**

This retrospective review evaluated 100 consecutive patients with psoriasis receiving anti-IL-17A therapies who were checked with at least twice QFT-GIT between 2016 and 2019 in National Taiwan University Hospital, Taipei and Hsin-Chu, Taiwan.

**Results:**

Among the 100 patients, the baseline QFT-GIT results were negative in 81.0% (81/100), positive in 18.0% (18/100), and indeterminate in 1.0% (1/100) of patients. The overall outcomes in patients receiving at least 6 months of cumulative exposure to anti-IL-17A agents were persistently seronegative in 80 patients (80.0%), persistently seropositive in 14 patients (14.0%), seroconversion in 1 patient (1.0%), seroreversion in 3 patients (3.0%), and others in 2 patients (2.0%). In patients with at least 11 months of cumulative exposure, the seroconversion rate was 1.3% (1/79). The only case with seroconversion had a positive QFT-GIT result previously. No case of TB reactivation or newly acquired TB infection was identified during the follow-up.

**Conclusions:**

In patients treated with anti-IL-17A monoclonal antibodies for psoriasis, routine serial repeat QFT-GIT testing was associated with lower seroconversion rate compared to real-world data of tumor necrosis factor-α inhibitors and anti-IL-12/23 antibody in Taiwan and in pivotal studies. Because clinical TB symptoms and signs are often preceded by QFT-GIF seroconversion, this result further supports the safety of anti-IL-17A agents in patients with psoriasis for LTBI.

## Introduction

For moderate to severe psoriasis, several monoclonal antibodies have been approved for the treatment, including tumor necrosis factor-α (TNF-α) inhibitors, an anti-interleukin (IL)-12/23 antibody, anti-IL-23 agents, an IL-17 receptor blocker, and IL-17A inhibitors. Secukinumab is a fully human IgG1 monoclonal antibody against IL-17A that selectively binds with the interleukin IL-17A cytokine and inhibits its interaction with IL-17 receptor; Ixekizumab is a humanized IgG4 monoclonal antibody that selectively binds with the interleukin IL-17A cytokine and inhibits its interaction with the IL-17 receptor. [1] The choice of drugs depends on the efficacy, risks, comorbidities, convenience, and cost.

Though generally safe, the administration of biologics often raises the concern of infection, including tuberculosis (TB), viral hepatitis [2–4] and pneumonia. TB, either reactivation of latent TB infection (LTBI) or newly acquired TB infection is an especially important safety issue due to the communicable nature and high prevalence in some countries. Despite the presence of many pivotal studies of the biologics, most of the studied were conducted in areas of low TB burden, [5] and cases of TB infection only appeared when the drugs are on the market in real-world practice. [6,7]

To detect LTBI, the whole blood interferon-γ release assays (IGRAs) and tuberculin skin test (TST) could be performed, with the former offering a better sensitivity (IGRAs: 89%, TST: 74%) and specificity (IGRAs: 98%, TST: 81%) profile, [8,9] especially in the population routinely receiving Bacilli Calmette-Guerin (BCG) vaccination. [10,11] Among the commercially available IGRAs, QuantiFERON-TB Gold In-Tube test (QFT-GIT; Cellestis Limited, Carnegie, Victoria, Australia) is more commonly applied.

In Taiwan, TB is not uncommon with Taiwan Centers for Disease Control reporting 11,528 cases of TB (49.4 cases per 100,000 populations) and 609 TB-related deaths in 2013. [12] For TNF-α inhibitors, screening and monitoring for LTBI before and during the treatment sessions are regarded as the standard of care due to the increased risk of LTBI reactivation. [13–18] The validity of IGRAs in screening LTBI before initiating TNF-α inhibitors had been well documented. [19–22] The risk of QFT-GIT tests positivity and seroconversion may be different according to the regions, ethnicities, comorbidities, concomitant medications, and underlying diseases. [23] Prior studies with serial QFT-GIT tests revealed an annual seroconversion rate between 0.38% to 18% in psoriatic patients receiving TNF-α inhibitors, [24–29] and the data from Taiwan was 14.29%. [30] Although routine prophylaxis use of isoniazid can significantly decrease the risk of LTBI, isoniazid resistance is not uncommon and may result in hepatotoxicity. Also drug compliance is another potential risk. Thus the pursuit of biologics with lower risk of LTBI is still needed.

Compared to TNF-α inhibitors, the newer biologics for psoriasis such as anti-IL-12/23 and anti-IL-17A agents are generally considered with a lower risk of active TB or LTBI reactivation. Nevertheless, for IL-12/23 inhibitor (ustekinumab), regular screening of LTBI during treatment is still listed on the guideline of several professional dermatologic associations. [31–33] Previous studies from Taiwan showed a lower QFT-GIT seroconversion rate (7.3%) during ustekinumab use compared to TNF-α antagonist (14.29%). [30,34] Although the presence of QFT-GIT seroconversion is not equal to clinical LTBI reactivation, QFT-GIT seroconversion usually precedes TB clinical symptoms and signs, and thus may be used as a surrogate markers for the inherent TB reactivation risks for biologics. Several case reports existed for the development of active TB after use of ustekinumab, and most of these cases came from the real-world data or post-marketing report. [35–37]

Compared to the anti-IL-12/23 agent, anti-IL-17A agents are generally considered to be safer in regard to latent TB reactivation or infection. [38] In a pooled analysis of 10 clinical studies in 3430 patients with moderate to severe plaque psoriasis treated with secukinumab, no case of reactivated LTBI was detected during the long-term follow-up. [39] Similarly, in one integrated safety analysis, no case of active TB was reported across 5730 patients with 13479 patient-years of exposure to ixekizumab. [40] Moreover, some experimental studies have demonstrated the lack of effect of secukinumab on *Mycobacterium tuberculosis* reactivation human *in vitro* model and animal model. [41,42]

However, there is limited real-world data providing the results of serial IRGAs among patients receiving anti-IL-17A therapies in TB intermediate or high burden countries. This study aims to evaluate the QFT-GIT status of psoriatic patients treated with secukinumab or ixekizumab in Taiwan.

## Materials and methods

### Patients

This study was approved by the Research Ethics Committee (REC) of National Taiwan University Hospital (NTUH) (201904124RINC) and NTUH, Hsin-Chu branch (105-064-E). We retrospectively enrolled consecutive psoriasis patients receiving anti-IL-17A therapies for at least 6 months and with at QFT-GIT check at least twice in NTUH, Taipei and Hsin-Chu, Taiwan from January 2014 to June 2019 on July 1^st^, 2019. The need for informed consent was waived by the REC of NTUH for the retrospective medical records prior to May 2019, and written informed consent was obtained from all the other patients. According to the local risk management plan in Taiwan, QFT-GIT has to be performed before biologic use followed by at least once yearly after treatment. Additional tests are usually performed during biologic switch and in the presence of clinical suspicion of TB infection, such as prolonged respiratory symptoms which are the most frequent adverse events during biologic use. [43] We defined the baseline QFT-GIT as the data checked on the nearest date to the initial exposure to anti-IL-17A therapies, and the follow-up QFT-GIT as the latest data checked during the follow-up. The number of months with exposure to anti-IL-17A agents in the timespan from the baseline to follow-up QFT-GIT was recorded. The patients without 6 months of overlapping period between the course of anti-IL-17A therapies and the timespan from baseline to follow-up QFT-GIT were excluded. We reviewed the medical records of these patients to evaluate clinical diagnosis, demographic information, medical history, and laboratory data.

The concomitant and immediately-before (defined as one month prior to the observed period) therapies along with anti-IL-17A therapies, which might have confounding effects, were recorded, including methotrexate, cyclosporine, sulfasalazine, leflunomide, nonsteroidal anti-inflammatory agents (NSAIDs), corticosteroid, tacrolimus, mycophenolate mofetil, and other target agents (etanercept, adalimumab, ustekinumab, golimumab, guselkumab, and tofacitinib). Also, the patients without exposure history to any kinds of biologics before the observed period were recorded as biologic-naïve.

### QuantiFERON-TB Gold In-Tube test for latent tuberculosis infection

QFT-GIT was performed in all the studied patients. Antigen with peptide cocktail simulating the proteins ESAT-6, CFP-10 and TB7.7 were used in the test. The interferon-γ (IFN-γ) values were calculated by subtracting the obtained value with nil antigens. A positive result was defined as IFN-γ ≥ 0.35 IU/ml and positive control value (IFN-γ of mitogen minus nil antigens) ≥ 0.5 IU/ml. A negative result was defined as 0 < IFN-γ < 0.35 IU/ml and positive control (mitogen) value ≥ 0.5 IU/ml. Indeterminate result was defined as IFN-γ of nil antigen > 8 IU/ml or positive control value < 0.5 IU/ml.

The final outcomes were defined according to the baseline and the follow-up QFT-GIT results during anti-IL-17A treatments: *seroconversion* as from negative to positive, *seroreversion* as from positive to negative, *persistently seronegative* as invariantly negative, *persistently seropositive* as invariantly positive, and *others* as any result showing indeterminate.

### Statistical analysis

Statistical analysis and graphs were done with standard spreadsheet software program using Microsoft Excel 2016 (Microsoft Corporation, Seattle, WA, USA). Fisher exact test was applied for groups comparison. Statistical significance was defined as p-value < 0.05.

## Results

### Patient characteristics

We enrolled 100 patients to this study in total (S1 Dataset). Of these patients, the mean age is 47.8 years [standard deviation (SD) 13.8 years], the male-to-female ratio is 74/26, and 40 patients (40.0%) also had psoriatic arthritis. The total observed period with anti-IL-17A exposure is 1254 person-months, with individual follow-up time varying from 6 to 24 months (median 12.5 months; mean 12.5 months, SD 4.0 months). The cumulative treated months were at least 11 months in 79 patients (79.0%). Most of the patients (96.0%) included in this study received only secukinumab as the anti-IL-17A agent, with the rest receiving ixekizumab only or switched agents between secukinumab and ixekizumab (due to inadequate or loss of efficacy to secukinumab). Thirty-seven patients (37.0%) and 67 patients (67.0%) were treated with concomitant and immediately-before therapies respectively, with NSAIDs (n = 19, 19.0%) and methotrexate (n = 32, 32.0%) as the most frequent medications. Twenty-two patients (22.0%) were biologic-naïve. The demographic features are presented in Table 1.

**Table 1:** Demographic features and final outcomes.

Number	100
Age (years), mean ± SD^†^	47.8 ± 13.8
Sex	
Male, n (%)	74 (74.0)
Female, n (%)	26 (26.0)
With psoriatic arthritis, n (%)	40 (40.0)
Anti-interleukin (IL)-17A agents	
Secukinumab, n (%)	96 (96.0)
Ixekizumab, n (%)	1 (1.0)
Switched, n (%)	3 (3.0)
With concomitant treatment, n (%)	37 (37.0)
With immediately-before treatment, n (%)	67 (67.0)
Biologic-naïve, n (%)	22 (22.0)
Cumulative exposure time to anti-IL-17A agents	
Total patient-months	1254
Median, months	12.5
Mean ± SD, months	12.5 ± 4.0
≥ 11 months, n (%)	79 (79.0)
Final outcomes	
Persistently seronegative, n (%)	80 (80.0)
Persistently seropositive, n (%)	14 (14.0)
Seroconversion, n (%)	1 (1.0)
Seroreversion, n (%)	3 (3.0)
Others, n (%)	2 (2.0)

^†^Standard deviation

### QuantiFERON-TB Gold In-Tube test results and final outcomes

**Fig 1:**
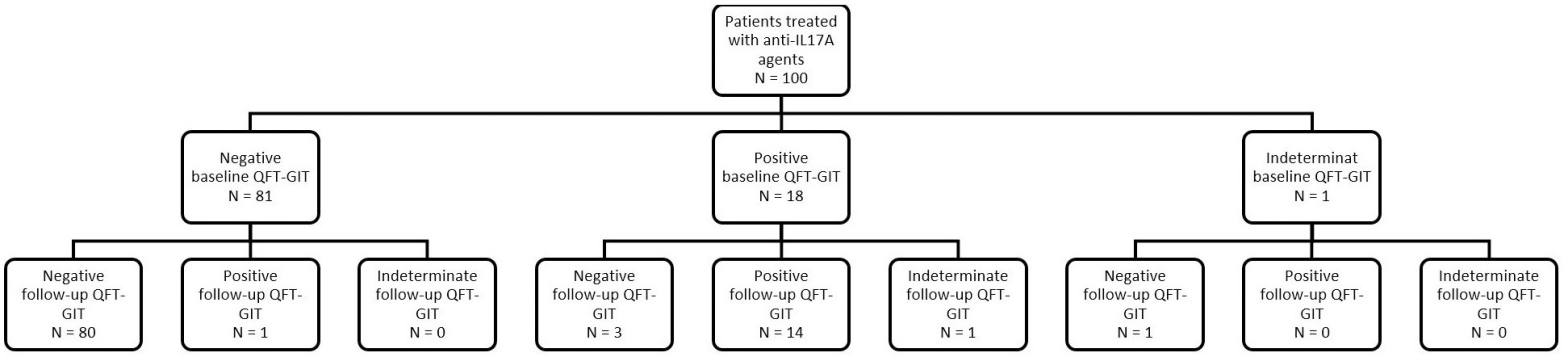
Results of QuantiFERON-TB Gold In-Tube test.

**Fig 2:**
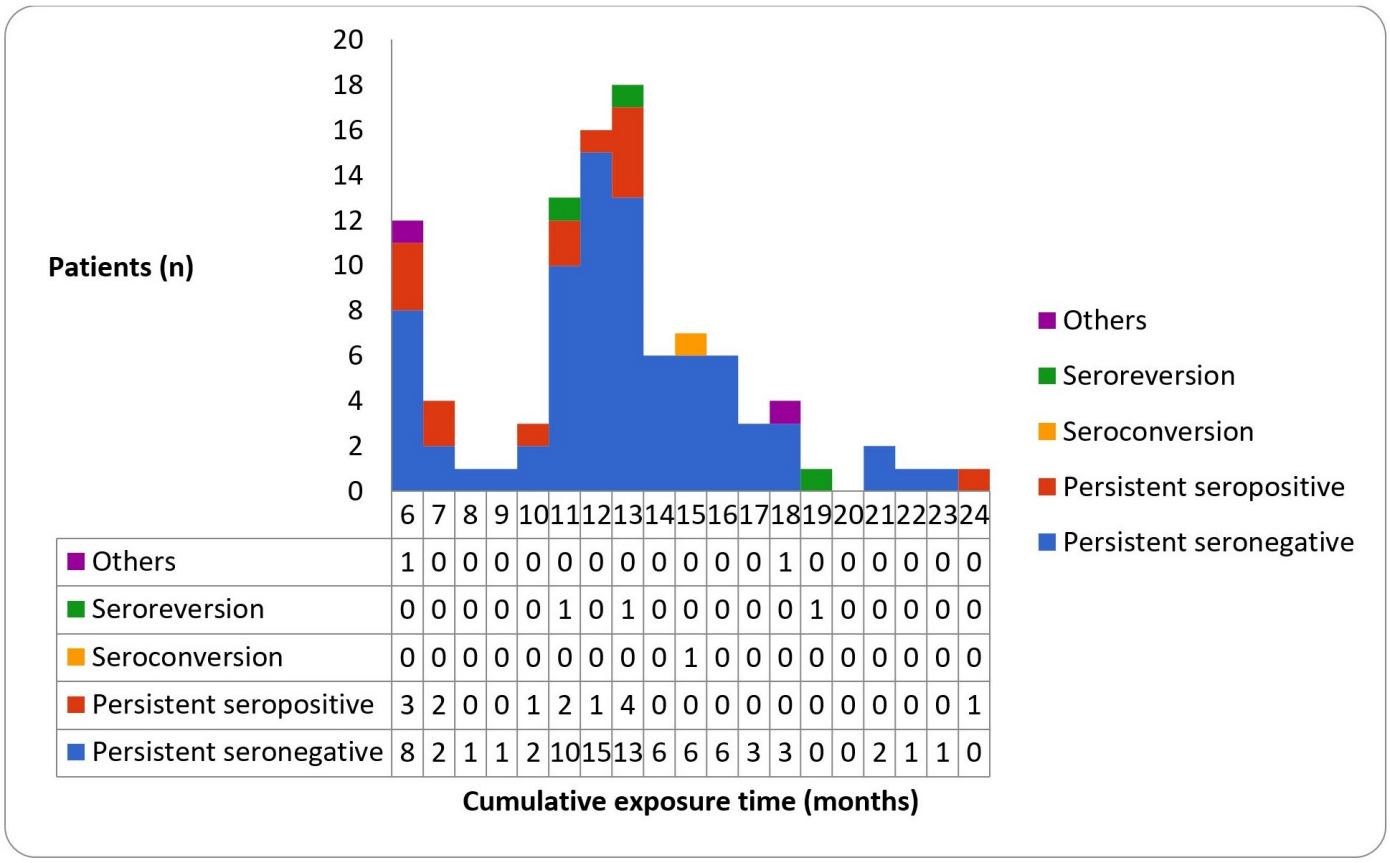
Final outcomes corresponding to the cumulative exposure time.

The final outcomes were summarized in Table 1. Patients flow-chart with results of baseline and follow-up QFT-GIT (Fig 1) and histogram with corresponding cumulative exposure time (Fig 2) were also shown. All the patients had a baseline QFT-GIT, with 81 patients (81.0%) showing negative, 18 patients (18.0%) showing positive, and 1 patient (1.0%) showing indeterminate results. All the patients with positive baseline QFT-GIT result had completed isoniazid (INH) prophylaxis therapy for LTBI in prior treatment courses. The follow-up QFT-GIT was measured at variable timing from 6 to 24 months for each patient, and the overall final outcomes were persistently seronegative in 80 patients (80.0%), persistently seropositive in 14 patients (14.0%), seroconversion in 1 patient (1.0%, details described in Table 2), seroreversion in 3 patients (3.0%), and others in 2 patients (2.0%). In patients with at least 11 months of follow-up time, the seroconversion rate was 1.3% (1/79). No case of TB reactivation or newly acquired TB infection was identified during the follow-up.

**Table 2:** Description of the patient with seroconversion outcome.

Age	54
Sex	Male
Disease status	Plaque psoriasis without psoriatic arthritis
Anti-interleukin (IL)-17A agent	Secukinumab
Concomitant treatment	Nil
Immediately-before treatment	Ustekinumab
Prior biologics	Ustekinumab
Cumulative exposure time to anti-IL-17A	15 months
Baseline interferon-γ value, IU/ml	0.23
Follow-up interferon-γ value, IU/ml	2.01

To identify the differences between the persistently seronegative group and seroconversion group, we divided the patients according to (I) whether receiving concomitant treatment (II) whether receiving immediately-before treatment (III) whether being biologic naive. No statistically significant difference was noted upon these analyses (Table 3).

**Table 3:** Comparison between persistently seronegative group and seroconversion group.

	Persistently seronegative, n	Sero-conversion, n		Persistently seronegative, n	Sero-conversion, n	p-value
With concomitant treatment	27	0	Without concomitant treatment	53	1	1
With immediately-before treatment	55	1	Without immediately-before treatment	25	0	1
Biologic-naïve	18	0	Non biologic-naïve	62	1	1

## Discussion

IL-17 is the key downstream cytokine driving psoriasis. [44] Treatment with anti-IL-17A monoclonal antibodies including secukinumab and ixekizumab leads to a rapid and robust clearing of psoriasis. Though inhibiting critical immune mediators may carry an increased risk of infections, the safety profile of anti-IL-17A therapies is generally favorable except for higher Candida infection and inflammatory bowel disease. [45–48]

Compared with TNFα inhibitors, the effect of IL-17A inhibition remains favorable in the host defense to TB infections. [49–53] IL-17 inhibitors have been recommended as one of the preferred agents in patients with LTBI. [38] A recent study showed that IL-17A actually promotes intracellular growth of *M*. *tuberculosis* by inhibiting apoptosis of infected macrophages. [54] Hence, anti-IL-17A treatment may limit intracellular growth of *M*. *tuberculosis* by enhancing apoptosis of infected macrophages.

The diagnosis of LTBI was defined as a positive QFT-GIT result and a negative chest X-ray or microbiological assay. The recommendation of tuberculosis screening was originally based on anti-TNF-α agents risk management plan during treatment of rheumatoid arthritis. At least 4-week treatment with isoniazid (INH) before anti-TNF-α agent use was suggested in cases of LTBI detected by either IRGAs or TST; a total 9-month INH is needed for complete treatment of LTBI. Due to the relatively lower TB risk of other monoclonal antibodies, including anti-IL-17A agents, a recently revised AAD-NPF guideline has suggested pre-treatment screening and subsequent yearly testing for latent TB only for patients with high risk and at the discretion of the dermatologist. [55] However, routine pre-treatment LTBI and subsequent yearly testing for latent TB is still often practiced or recommended. [32,33,56]

Reviewing the history of the only patient with QFT-GIT serooconversion in our study (Table 2), an episode of seroconversion (IFN-γ value to 2.23 IU/ml) was present already in his previous treatment course of ustekinumab. He received a 9-month INH therapy for LTBI, and the IFN-γ value decreased to 0.23 IU/ml in the follow-up. Yet another serooconversion with IFN-γ value elevating to 2.01 IU/ml was noted after use of secukinumab for 15 months. Chest X-ray and sputum culture revealed negative results. Since IGRA could be persistently positive in the majority of patients after the INH therapy, [57] and a completed course of treatment is usually sufficient for LTBI, close observation was suggested by the infection specialist. In fact, the routine use of INH prophylaxis based only on positive QFT-GIT result is arguable due to the risk of INH resistance and INH toxicity. [58,59] Meanwhile, there was no statistically significant difference in whether receiving concomitant or immediately-before treatments, and whether being biologic-naïve between the seroconversion group and the persistently seronegative group in our study (Table 3). As for the persistently seropositive group, all the cases (14/14) in our study had received INH therapy previously and the subsequent treatment with anti-IL-17A agents did not show evidence of TB reactivation during the follow-up.

In psoriatic patients treated with anti-IL-17A agents, a low QFT-GIT tests seroconversion rate was demonstrated in our study. The increased seroconversion rate in the cohort with longer follow-up time (11 months) resulted from a fewer population rather than more cases. No case of TB reactivation or newly acquired TB infection was identified during the observed period in our study. This real-world data supported the findings in prior clinical studies showing no evidence of increased *M*. *tuberculosis* infections under anti-IL-17A therapies.

In conclusion, we found routine serial repeat QFT-GIT testing in patients using anti-IL-17A monoclonal antibodies for psoriasis was associated with very low seroconversion rate in Taiwan, compared to tumor necrosis factor-α (TNF-α) inhibitors (14.29%) [30] and ustekinumab, an anti-interleukin (IL)-12/23 antibody (7.3%). [34] Though the absence of QFT-GIT seroconversion could not completely rule out TB infection, [60] the anti-IL17A antibodies may carry the lowest TB risk for psoriatic patients, compared to other biologics. We also recommend the use of clinical vigilance of relying more on clinical symptoms, exposure and travel history for the monitor of TB during treatment of psoriasis. Our study is limited by the limited subjects, retrospective nature and the short follow up periods. Additional studies from controlled studies in higher TB burden countries are needed.

## Supporting information

S1 Dataset Primary data of enrolled patients. (XLSX)
